# Perioperative volume management for esophageal cancer surgery

**DOI:** 10.1186/cc12132

**Published:** 2013-03-19

**Authors:** M Karaman Ilic, G Madžarac, J Kogler, D Stančić Rokotov, N Hodoba

**Affiliations:** 1 University Hospital Centre Zagreb, Croatia

## Introduction

Pulmonary complications are the primary reason for extending a patient's stay in the ICU. The aim of this study was to investigate whether perioperative volume management can influence the PaO_2_/FiO_2 _value and total length of stay in the ICU.

## Methods

Sixteen patients were divided into two groups: one group was treated with a restrictive approach (≤8 ml/kg/hour), and the other with a liberal approach (> 8 ml/kg/hour). Patients were randomly allocated using sealed envelopes. During the thoracic part of the surgical procedure, all patients received one-lung ventilation (OLV).

## Results

In the group treated with a restrictive volume approach, patients received fluids at the rate of 7.0 ± 1.0 ml/kg/hour. PaO_2_/FiO_2 _was 288 ± 14 after intubation and 270 ± 22 before extubation. In the group treated with a liberal volume approach, fluids were replaced at 11.0 ± 2.0 ml/kg/hour. PaO_2_/FiO_2 _was 259 ± 24 after intubation and 223 ± 43 before extubation. Surgery combined with OLV was found to significantly affect the PaO_2_/FiO_2 _value (ANOVA, *F*_1,14 _= 15.85a, *P *= 0.001, partial η^2 ^= 0.531). The average PaO_2_/FiO_2 _level was significantly higher in the restrictive-replacement group than in the liberal-replacement group (ANOVA, *F*_1,14 _= 9.66, *P *= 0.008, partial η^2 ^= 0.408). There was no interaction between the groups (ANOVA, *F*_1,14 _= 1.7a, *P *= 0.215, partial η^2 ^= 0.108). Mean length of stay in the ICU was similar between the restrictive-replacement group (5.2 ± 2.3 days) and the liberalreplacement group (6.3 ± 1.6 days) (ANOVA, *F*_1,14 _= 0.814a, *P *= 0.382, partial η^2 ^= 0.055).

## Conclusion

Results from this small sample indicate that esophageal carcinoma surgery by itself had a detrimental effect on the PaO_2_/FiO_2 _value, which restriction of perioperative volume did not significantly affect. Volume restriction also did not affect length of stay in the ICU.
